# Dual Role of Autophagy in Diseases of the Central Nervous System

**DOI:** 10.3389/fncel.2019.00196

**Published:** 2019-05-28

**Authors:** Tamara Bar-Yosef, Odeya Damri, Galila Agam

**Affiliations:** Department of Clinical Biochemistry and Pharmacology and Psychiatry Research Unit, Faculty of Health Sciences, Ben-Gurion University of the Negev and Mental Health Center, Beersheba, Israel

**Keywords:** autophagy, central nervous system, neurodegenerative diseases, psychiatric disorders, lithium

## Abstract

Autophagy is a vital lysosomal degradation and recycling pathway in the eukaryotic cell, responsible for maintaining an intricate balance between cell survival and cell death, necessary for neuronal survival and function. This dual role played by autophagy raises the question whether this process is a protective or a destructive pathway, the contributor of neuronal cell death or a failed attempt to repair aberrant processes? Deregulated autophagy at different steps of the pathway, whether excessive or downregulated, has been proposed to be associated with neurodegenerative disorders such as Alzheimer’s-, Huntington’s-, and Parkinson’s-disease, known for their intracellular accumulation of protein aggregates. Recent observations of impaired autophagy also appeared in psychiatric disorders such as schizophrenia and bipolar disorder suggesting an additional contribution to the pathophysiology of mental illness. Here we review the current understanding of autophagy’s role in various neuropsychiatric disorders and, hitherto, the prevailing new potential autophagy-related therapeutic strategies for their treatment.

## Introduction

Autophagy is an essential process by which cytoplasmic molecules (proteins, lipids, nucleic acids, and polysaccharides) (Farre and Subramani, 2004; Uttenweiler and Mayer, 2008) and subcellular organelles such as mitochondria (mitophagy) (Farre et al., 2009; Kiel, 2010) are delivered to the lysosome for degradation and recycling. It is an evolutionarily conserved pathway occurring in eukaryotic cells. It is induced under stress conditions such as absence of growth factors, low oxygen levels, or nutrient starvation (Levine and Klionsky, 2004; Wang et al., 2006; Williams et al., 2006; Mazure and Pouyssegur, 2010) assuring cell survival. It is also required for development, differentiation, homeostasis and, in specific conditions, cell death (Levine and Kroemer, 2008; Martinet et al., 2009; Wu et al., 2015). The three forms of autophagy are: macroautophagy – the main pathway designated autophagy, microautophagy, and chaperone-mediated autophagy (CMA; Hu et al., 2015; Wu et al., 2015). In autophagy cytosolic double-membrane structures – autophagosomes – are formed. They sequester protein aggregates, damaged organelles, and microbes (Perrotta et al., 2015), moving along microtubules toward microtubule-organizing center (Jahreiss et al., 2008), subsequently fusing with the lysosome to form autolysosomes where degradation and recycling by acidic lysosomal hydrolases takes place (Wang et al., 2006; Puyal et al., 2012).

The main autophagy signaling is regulated by the mTOR pathway, essential for cell growth and protein synthesis among many other cellular pathways (Guertin and Sabatini, 2009). There are two mTOR complexes: mTOR complex 1 (mTORC1) and mTOR complex 2 (mTORC2) (Guertin and Sabatini, 2009). The mechanism(s) via which mTORC1 regulates autophagy are (is) currently unclear (Thoreen et al., 2009). In response to growth factors signaling (insulin in particular) and to elevated nutrient levels, conditions which inhibit AMP-dependent kinase (AMPK; Meijer and Codogno, 2006; Cheong and Klionsky, 2014) mTOR suppresses autophagy. The most known pathway of upregulating autophagy is via the lipophilic antibiotic rapamycin, inactivating mTOR. Down-regulation of mTOR through rapamycin prevented mitochondrial-dependent apoptosis and resulted in the activation of autophagy (Ravikumar et al., 2004; Eisenberg-Lerner et al., 2009; Nikoletopoulou et al., 2013). Pre-treatment with rapamycin has been shown to protect cell lines from pro-apoptotic insults in an autophagy-dependent way (Ravikumar et al., 2006). These protective effects suggest that autophagy may protect from cell death (Williams et al., 2006). However, rapamycin treatment does not affect autophagosome dynamics (movement), so clearance by autophagy induction is likely because of the increase in autophagosome formation (Jahreiss et al., 2008). Others autophagy mediators include the autophagy-related genes (ATG), conserved from yeast to mammals, and the microtubule-associated protein 1A/1B-light chain 3 (LC3), a protein family comprised of LC3, LC3A, and LC3B. During autophagosomes engulfment LC3 is cleaved to LC3-I and converted by phosphatidylethanolamine to LC3-II. The latter is recruited to autophagosomal membranes (Tanida et al., 2008). LC3-II, a mammalian homolog of Atg8, is the best known autophagosome-specific protein marker (Mizushima, 2007) presented inside and outside of the autophagosomes. Its amount correlates with the size of the autophagosomes (Kabeya et al., 2000). p62/sequestosome 1 is a protein complex that when binding with LC3-interacting region (LIR; Lim et al., 2011) can localize to autophagic compartments transporting ubiquitinated proteins for degradation. This process is important for autophagic vacuoles (AVs) containing specific cargo (Kirkin et al., 2009). The protein Beclin1 is another regulator (the mammalian ortholog of the yeast Atg6/Vps30 gene). It interacts with B-cell lymphoma 2 (Bcl-2)-like proteins to form a complex. The complex is a key regulator of autophagy. Namely, its modulation either stimulates or inhibits this cellular pathway (Cao and Klionsky, 2007; He and Levine, 2010). In microautophagy cytoplasmic content is delivered into the lysosome by invagination of the lysosomal membrane into its lumen. Overall, the molecular mechanism of microautophagy regulation is still unclear (Hu et al., 2015). In the CMA pathway, activated during cellular hypoxic and ischemic stress, chaperones selectively transfer cytosolic protein aggregates into the lysosome for degradation. This occurs without vesicle formation (Dohi et al., 2012; Wu et al., 2015).

Autophagy dysfunction has previously been associated with a variety of diseases such as cancer (Gozuacik and Kimchi, 2004), muscular disorders (Danon’s disease) (Nishino et al., 2000), pathogen infections (Listeria monocytogenes) (Shintani and Klionsky, 2004), cerebral ischemia (Wei et al., 2012), neurodegeneration (Wong and Cuervo, 2010; Puyal et al., 2012; Wei et al., 2012), psychiatric disorders (Kara et al., 2013; Jia and Le, 2015; Yuan et al., 2015), and more. This review will focus on autophagy and its dual role – in cell survival and in cell death in the central nervous system (CNS), namely, in neurodegenerative [Alzheimer’s disease (AD), Parkinson’s disease (PD), and Huntington’s disease (HD) (Wu et al., 2015)] and psychiatric [schizophrenia (Yuan et al., 2015), bipolar disorder (BPD) (O’Shea and McInnis, 2015), and unipolar depression (Jia and Le, 2015)].

Post-mortem brain studies which found accumulation of autophagosome-like vesicles in axonal terminals of dysfunctional or degenerating neurons (Yue, 2014) in neurodegenerative disease patients (Yamamoto and Yue, 2014), deviated expression of autophagy-related proteins in schizophrenia patients (Merenlender-Wagner et al., 2014) along with studies indicating that autophagy enhancers induce an anti-depressant-like effect (Cleary et al., 2008; Kara et al., 2013; Jia and Le, 2015) implicate the involvement of aberrant autophagy in the pathogenesis of these disorders and suggest that enhancing the process protects against them (Levine and Kroemer, 2008).

## Autophagy and Neurodegenerative Disorders

Autophagy in the CNS is constitutively active (Komatsu et al., 2006), maintaining an important housekeeping role by eliminating defective proteins and organelles, preventing the accumulation of aggregates, providing energy demands, and supporting neuronal plasticity (Wang et al., 2006; Fimia et al., 2007; Komatsu et al., 2007). Evidence indicates that autophagy plays a neuroprotective role (Puyal et al., 2012). This role is particularly important in post-mitotic cells including neurons in order to protect against neurodegeneration and to promote cell survival (Levine and Kroemer, 2008; Nikoletopoulou et al., 2013). The neuronal soma exhibits basal levels of autophagy, mitophagy, and CMA, maintaining cellular homeostasis. Axon injuries induce accumulation of autophagosomes or AVs and dystrophic axonal swellings. Synapses require high energy and protein turnover. Therefore they are more vulnerable to autophagy dysfunction (Son et al., 2012).

On the other hand, autophagy has been associated with promotion of cell death (type-II cell death) as a result of circumstances such as excessive activation of autophagy [e.g., overexpression of the Atg1 in Drosophila (Scott et al., 2007), requirement for massive cell elimination and neuronal excitotoxicity (Clarke, 1990; Komatsu et al., 2006; Ginet et al., 2009)]. It is possible that excessive up-regulation of autophagy and long-term autophagy up-regulation eventually result in self-digestion or have harmful effects (Martinet et al., 2009; Renna et al., 2010; Puyal et al., 2012). The role of autophagy in cell death and the detailed mechanism are still unclear and it is under debate whether type-II cell death is apoptosis-related or is a separate process (Kroemer and Levine, 2008; Levine and Kroemer, 2008; Puyal and Clarke, 2009). Interestingly, apoptosis and autophagy share regulators such as Beclin1, Bcl-2, p53, and Atg5 which may interact to promote neuronal cell death (Xue et al., 1999; Mizushima et al., 2008). Beclin1 has been linked to protection against neurodegenerative diseases and to lifespan extension (He and Levine, 2010).

Protein aggregates are a result of defective protein production that can be caused by oxidative stress, ultraviolet radiation, toxic compounds, genetic mutations, post-translational modifications and more (Markossian and Kurganov, 2004). The soluble forms are targeted for degradation via the CMA or the ubiquitin-proteasome pathways (UPS). The insoluble forms of the proteins form complex structures (oligomers and fibrils) making it difficult to traverse through the proteasome’s pore (Verhoef et al., 2002) or to be degraded by the CMA pathway (Levine and Kroemer, 2008). In these instances autophagy is activated (Rubinsztein et al., 2005). However, when autophagy and other protein degradation systems are impaired/altered, neurons display accumulation of defective or mutant protein aggregates (Ross and Poirier, 2004) leading to a toxic effect of cellular damage and ultimately to cell death associated with neurodegeneration (Hara et al., 2006; Komatsu et al., 2006; Levine and Kroemer, 2008; Nikoletopoulou et al., 2013). Indeed, blockade of autophagy in Atg5- and Atg7-deficient mice lead to the appearance of inclusion bodies accumulated in the cerebral cortex and the cerebellum, neurodegeneration and loss of neurons (Hara et al., 2006; Komatsu et al., 2006).

Presence of excessive accumulation of autophagy vesicles (autophagosomes and lysosomes) has been observed in post-mortem brain of patients with AD, PD and HD (Rubinsztein et al., 2005, 2007; Williams et al., 2006) raising the question whether the accumulation of these vesicles is a result of autophagy dysfunction (Martinez-Vicente and Cuervo, 2007; Rubinsztein et al., 2007) or due to excessive autophagy (Shintani and Klionsky, 2004).

### Alzheimer’s Disease

Alzheimer’s disease, the most common neurodegenerative disorder (Alzheimer’s, 2016; Liu et al., 2018), is defined by memory loss and a decline in cognitive functions (Terry and Davies, 1980). It is characterized by progressive loss of neurons due to two pathologic lesions: (i) senile plaques consisting of extracellular β-amyloid (Aβ) deposit scattered among axons and dendrites. The plaques are derived from amyloid-precursor protein (APP) proteolysis (Yu et al., 2005; Martinet et al., 2009) with an unclear biological function (Nixon, 2007); (ii) neurofibrillary tangles composed mainly of tau-proteins that are abnormally phosphorylated and aggregated into filaments inside the neurons (Nixon, 2007).

As recently reviewed (Li et al., 2017; Moloudizargari et al., 2017) dysfunctional autophagy is implied in AD’s pathogenesis. Evidence shows impaired maturation or fusion of autophagosomes with lysosomes or their transport toward the neuronal cell body. Endosomal–lysosomal dysfunction has also been presented as accumulation of AVs containing Aβ within dystrophic neurons and dendrites (neurites) in post-mortem human brain and in a mouse model of AD (Nixon et al., 2005; Yu et al., 2005). It is unclear whether this reflects defective AVs clearance due to impaired autophagosomes-lysosomal fusion or due to enhanced autophagy (Rubinsztein et al., 2005). Inhibition of dynein function in PC12 cells [dyneins are microtubule-based motor proteins responsible for the movement of cargo from the distal ends of axons to the cell bodies of neurons (Goldstein and Yang, 2000)] impaired the process and slowed clearance of proteins proned to aggregate (e.g., A53T α-synuclein) (Ravikumar et al., 2005). A study in mouse blastocysts has shown that a mutation in the presenilin-1 gene (PS1) caused lack of clearance by autophagy and might account, at least partly, for protein accumulation in AD. PS1 is required for lysosomal turnover of autophagic and endocytic protein substrates. PS1 deletion caused loss of autophagy function due to impaired autolysosome acidification and cathepsin activation [cysteine cathepsins are proteases responsible for proteolytic degradation within the lysosome (Stoka et al., 2016)] (Lee et al., 2010). Accordingly, enhancing lysosomal cathepsin activity in an AD mouse model reduced the accumulation of Aβ, ubiquitinated proteins and other autophagic substrates within autolysosomes-lysosomes, and decreased extracellular and total brain amyloid deposition (Yang et al., 2011). Overall, these studies present prominent lysosomal dysfunction in AD (Whyte et al., 2017). In AD mice models autophagosome presence in dendrites increases before deposits are created, indicating autophagy as a primary response and not as an outcome of deposit formation (Nixon, 2007). It is suggested to be a prior event of the abnormally accumulation of AV’s. Furthermore, APP, PS1 and other substrates necessary for the generation of Aβ peptides were identified in isolated AVs of AD mice models livers (Yu et al., 2004), suggesting potential AVs’ role in Aβ generation (Yu et al., 2005).

Long-term inhibition of mTOR by rapamycin administration (to increase autophagy) prevented AD-like cognitive deficits and lowered Aβ levels (Spilman et al., 2010). A study in AD patients’ mid-frontal cortex gray matter found reduction of beclin1 protein levels. Inducing haploinsufficiency of the beclin1 gene in mice decreased neuronal autophagy and led to neurodegeneration and lysosome disturbance. Moreover, in an AD mouse model, reduction of beclin1 expression resulted in increased intraneuronal and extracellular Aβ accumulation, substantial neuronal abnormalities and neurodegeneration (Pickford et al., 2008). According to evidence presented above of gradually impaired autophagy seen as autophagic-lysosomal dysfunction and the loss of proteasome activity (Keller et al., 2000), neurons are left without a coping mechanism against toxic protein accumulation and there is a sense in restoring normal autophagy as a therapeutic strategy.

A vicious circle, in which neuronal mitochondrial dysfunction, apparently due to impaired mitophagy occurring early in the disease development and resulting in oxidative damage and cellular energy deficits which, in turn, further impair mitophagy has also been recently suggested to promote Aβ and Tau pathologies (Simoncini et al., 2015).

### Huntington’s Disease

Huntington***’***s disease is an inherited autosomal dominant disease (Martin and Gusella, 1986) characterized by insoluble aggregation of an abnormally long polyglutamine (also called inclusions) caused by a gain-of-function mutation in the huntingtin gene (The Huntington’s Disease Collaborative Research Group, 1993; Gil and Rego, 2008). This accumulation leads to progressive memory loss, cognitive decline, impaired movement and more (Martin and Gusella, 1986). The mutation is expressed by expansion of cytosine–adenine–guanine (CAG) repeats at the N-terminus of the huntingtin gene. This condition contributes to slow progressive neuronal loss in the striatum and cortex that leads to death. The onset age is between 35 and 50 years and it is a progressive disease with clinical manifestations such as motor disturbances, cognitive decline, bradykinesia, rigidity, muscle wasting, weight loss, and eventually death (Gil and Rego, 2008). Diverse mechanisms such as apoptosis, oxidative stress and mitochondrial dysfunction have been implicated in HD pathophysiology (Gil and Rego, 2008). In addition, studies suggest that autophagy plays a central role in the progressive degeneration of HD (Martinet et al., 2009). Indeed, observations in HD mouse models and in cells from humans with HD identified a specific autophagic defect in which the ability of AVs to recognize and trap cytosolic cargo is compromized (Martinez-Vicente et al., 2010).

Huntington’s disease mouse models show that the ubiquitin-proteasome system tends to become overloaded with the increasing number of aggregates and this impairment activates cell death pathways through apoptosis or autophagy (Iwata et al., 2005). Expression of mutated huntingtin causes endosomal-lysosomal activity (Kegel et al., 2000) and HD brains display endosomal-lysosomal organelle accumulation (Tellez-Nagel et al., 1974; Sapp et al., 1999). Also, it has been shown that the number of AVs in lymphoblasts of HD patients correlated with the length of the polyglutamine expansion (Nagata et al., 2004).

Autophagy may be the cell’s way to eliminate the huntingtin mutants. It has been reported that abolishing huntingtin mutant expression in mice models of HD prevents symptoms of progression and may reverse aggregate formation and progressive motor decline (Yamamoto et al., 2000). Also, stimulation of autophagy in clonal striatal cells, PC12 cells and rodent embryonic cells promoted degradation of huntingtin whereas blocking autophagy reduced cell viability and increased the number of cells containing aggregates of mutant huntingtin (Qin et al., 2003). Over-expression of the transcription factor TFEB (transcription factor EB) in the striatum of HD-mutant mice stimulated autophagy and lysosome activity and lowered mutant huntigtin levels (Vodicka et al., 2016). Another study supporting this observation has shown increased autophagy only in HD model cells expressing mutant aggregates and demonstrated that treatment with rapamycin or its analog CCI-779 in fly and mouse models reduced aggregate formation and cell death and presented improvements in phenotypes related to neurological dysfunction (Ravikumar et al., 2004). Yet, rapamycin’s mTOR activity inhibition might be impaired following long huntingtin expression or increased formation of its aggregates probably due to mTOR sequestration in inclusions resulting in lower levels of soluble mTOR (Ravikumar et al., 2002, 2004).

Studies have revealed that if the production of the aggregates is halted the cells have the ability to clear them. In a study that exemplifies this notion autophagy triggered clearance through the activation of beclin1 and Vps34 promoted by insulin receptor substrate 2, despite activation of Akt and mTOR. This implies an mTOR-independent autophagy activation (Yamamoto et al., 2006). However, accumulated mutant huntingtin recruits Beclin1 impairing its activity thereby reducing autophagic degradation. The mechanism of the latter process has recently been uncovered by Ashkenazi et al. (2017). They demonstrated that the activity of ataxin-3 (which interacts with beclin1 allowing its deubiquitinase activity to protect beclin1 from proteasome-mediated degradation enabling autophagy) mediated by its polyQ domain was competed by other soluble proteins with polyQ tracts in a length-dependent fashion. In cells expressing mutant huntingtin exon 1, in brain of HD mouse model and in HD patient cells this resulted in impaired starvation-induced autophagy. They also observed decreased beclin1 levels and impaired starvation-induced autophagy in fibroblasts derived from HD patients, compared to controls. Furthermore, in human brains beclin1’s expression declines with age which may result in parallel reduction in autophagic activity (Shibata et al., 2006).

### Parkinson’s Disease

Parkinson***’***s disease is characterized by tremors, muscular rigidity, and bradykinesia caused by progressive loss of dopaminergic neurons in the substantia nigra pars compacta (SNpc) followed by accumulation of mutant protein inclusions in Lewy bodies (Gelb et al., 1999). Interestingly, mutations in different subtypes of PD can affect different stages of autophagy (Karabiyik et al., 2017). In familial PD accumulation of mutant α-synuclein (A53T and A30P) in the Lewy bodies is implicated in the disease pathogenesis (Duda et al., 2002; Cuervo et al., 2004). Wild-type α-synuclein, a protein of unknown function, is implied as a pre-synaptic regulator of dopamine neurotransmission (Abeliovich et al., 2000) and is normally translocated into lysosomes for CMA degradation (Webb et al., 2003; Cuervo et al., 2004). The pathogenic α-synuclein mutants bind to the lamp2a receptor located on the lysosomal membrane acting as uptake blockers thereby inhibiting their own and other substrates’ degradation, resulting in an up regulation of autophagy as a compensatory response (Martinet et al., 2009). It has been shown that autophagy plays a role in the degradation of the α-synuclein mutants (Webb et al., 2003). Accordingly, lysosome-like vacuoles were observed in the dopaminergic neurons of the SNpc of PD patients (Anglade et al., 1997; Zhu et al., 2003). Inhibition of autophagy by α-synuclein over-expression has been shown to result in mislocalization of the autophagy protein Atg9 and to decrease autophagosome precursors formation (Winslow et al., 2010).

Another mutated gene that has been linked to PD is the loss-of-function Park2, coding for the ubiquitin-ligase parkin. In mammalian cells parkin is recruited to dysfunctional mitochondria exhibiting low membrane potential mediating the engulfment of mitochondria by autophagosomes. Mutations in this gene result in failure to eliminate dysfunctional mitochondria, implying autophagic and mitochondrial dysfunction in the pathogenesis of PD (Narendra et al., 2008). In a recent review Beilina and Cookson (Beilina and Cookson, 2016) discuss the relationships between protein products of PD genes and indicate their links with regulation of the autophagy–lysosome system attesting that such an interaction only exists for some of the genes. Similarly, Beneto et al. (2016) propose that the ATP13A2 gene (mutated in some types of early-onset Parkinsonism) and the SYT11 gene (associated with PD, the transcription of which is regulated by ATP13A2) comprize a regulation network of the autophagy–lysosome pathway. To sum-up, the autophagy–lysosome pathway in PD might be the neurons’ attempt to compensate dysfunctional degradation pathways that backfire, causing further damage to the cell, eventually leading toward cell death, thus contributing to PD-associated neurodegeneration. However, perplexing data have recently been described. Based on the report that microRNA (miR)-181a is downregulated in PD (Ding et al., 2016), Liu et al. (2017) overexpressed miR-181a in MPP-treated human neuroblastoma cells (an accepted PD-like model). It resulted in a significant decrease of the expression of the autophagy markers LC3II/LC3I ratio and Beclin1, but also in attenuated cell apoptosis.

## Autophagy and Psychiatric Disorders

### Schizophrenia

Schizophrenia is a mental disorder with a prevalence of 1% in the population characterized by positive (e.g., disturbed thoughts and perception, delusions, and hallucinations) and negative (e.g., diminished emotional expression and motivation) symptoms, disorganized thinking, cognitive and emotional decline, severe reduction in social functioning and hampered coping abilities (Perala et al., 2007; Rezin et al., 2009; Tandon et al., 2013; Yuan et al., 2015). The onset mostly occurs at late adolescence (Perala et al., 2007; Rezin et al., 2009). The etiology is unknown, but it appears to have developmental changes at synapses among other sites (Perala et al., 2007; Balu and Coyle, 2011). Autophagy is suggested to have a key role in the pathophysiology of schizophrenia (Merenlender-Wagner et al., 2014, 2015; Yuan et al., 2015). Beclin1 mRNA levels were reported to be 40% reduced in post-mortem hippocampal samples of schizophrenia patients compared to control subjects (Merenlender-Wagner et al., 2015). Similarly, reduced Beclin1 mRNA and protein levels and decreased LC3-II levels were found in the hippocampus of schizophrenia mouse models (Merenlender-Wagner et al., 2014; Yuan et al., 2015).

Schizophrenia, originally named “dementia praecox” and AD share similarities and it is suggested that neurodegeneration also plays a role in the pathophysiology of schizophrenia (Lieberman, 1999; Horesh et al., 2011). Both disorders share gene expression changes in the superior temporal gyrus including downregulation of autophagy-related genes such as beclin1 and ATG3 (Horesh et al., 2011). This may suggest that autophagy dysfunction is shared by both disorders, although their underlying neurobiology is different.

Activity-dependent neuroprotective protein (ADNP) is necessary for brain formation, development, neuroprotection, and plasticity. The protein directly interacts with LC3II, suggesting its role in autophagy (Bassan et al., 1999; Merenlender-Wagner et al., 2015). ADNP^+/−^mice exhibited cognitive dysfunction, increased tau hyper-phosphorylation and memory impairment, tangle-like structures and neurodegeneration reminiscent of AD (Vulih-Shultzman et al., 2007). Our group has demonstrated that “disturbance of ADNP in schizophrenia has a negative effect on autophagic activity; ADNP and ADNP2 (homologous protein of ADNP) expression was deregulated in post-mortem hippocampus of schizophrenia patients compared to healthy matched controls and the deregulation correlated with the disease progression” (Dresner et al., 2011; Merenlender-Wagner et al., 2015). We argued that “increased ADNP and ADNP2 expression in lymphocytes from schizophrenia patients compared to healthy controls and a negative correlation with disease duration may be a compensatory mechanism. Additionally, beclin1 mRNA levels was decreased by ∼20% in the ADNP^+/−^ mouse hippocampus, coupled to ∼30% increase in Bcl2, suggesting an impact of ADNP deregulation on brain autophagy” (Merenlender-Wagner et al., 2015). Interestingly, administration of the peptide sequence NAP (NAPVSIPQ), derived from ADNP, inhibited apoptosis (Leker et al., 2002; Gozes and Divinski, 2007), enhanced ADNP-LC3-II interaction (Merenlender-Wagner et al., 2014), reversed the decrease in hippocampal Beclin1 mRNA levels in a mouse model of schizophrenia (MAP6-deficiency) and decreased cognitive deficits and tau hyperphosphorylation in ADNP^+/−^mice (Vulih-Shultzman et al., 2007). NAP also decreased schizophrenia-like hyperactivity in a different mouse model of schizophrenia (Merenlender-Wagner et al., 2010). Phencyclidine (PCP), a non-competitive antagonist of glutamatergic *N*-methyl-D-aspartate receptor, induces positive- and negative-like signs exhibited by schizophrenia patients (Javitt et al., 2012).

Recently, Jevtic et al. (2016) used the PCP-induced schizophrenia-like behavior in perinatal rats and reported alterations in autophagy markers.

Another indication for the possible involvement of aberrant autophagy in schizophrenia relates to the apolipoprotein L1 (ApoL1) gene. Overexpression of ApoL1 induces autophagic cell death (Zhaorigetu et al., 2008) and expression of ApoL1 was found to be significantly upregulated in post-mortem brain of schizophrenia patients (Mimmack et al., 2002). Moreover, in humans, the ApoL1 gene is localized to chromosome 22q13.1, a region with loci suggested to be linked to susceptibility to schizophrenia (Takahashi et al., 2008). Observation of reduced Beclin1 in post-mortem brains of schizophrenia patients and mouse models could indicate autophagy’s link to the pathogenesis of the illness.

### Bipolar Disorder

Bipolar disorders (Belmaker, 2004) are a group of mental disorders with high heritability (McGuffin et al., 2003; Kieseppa et al., 2004) and estimated life-time risk of 1–2% in the adult population. BPDs are characterized by mood swings between depression and mania (Manji and Lenox, 1998) resulting in hampered functioning, impaired cognition and disrupted life of the patients and their families (Zarate et al., 2006a. The manic phase is defined by a “hyper” state, enhanced motor activity, improper judgment, racing thoughts and decreased sleep. The depressive phase is defined by major depression symptoms, such as depressed mood, cognitive changes, psychomotor changes, and more (Goodwin and Jamison, 1990; Gould et al., 2004). The etiology/pathophysiology of the illnesses is still unknown but accumulating data point at brain mitochondrial dysfunction as a central characteristic of BPDs (Scaini et al., 2016). Toker and Agam (2015) suggested that disturbed mitochondrial function in BPD is a result of impaired autophagy.

Searching for studies linking autophagy with BPD yielded very few indirect results, dealing, mostly, with mood stabilizers, and autophagy (see section “The potential of autophagy-related therapeutic strategies for the treatment of neurodegenerative and psychiatric disorders”).

### Major Depression Disorder

Major depressive disorder (MDD) is a mental illness characterized by depressed mood, decreased interest or pleasure in most activities, low energy or fatigue, sleep disturbances and more (Belmaker and Agam, 2008; Eshel and Roiser, 2010; American Psychiatric Association, 2016). This condition has an overall lifetime prevalence of up to 17% (Larsen et al., 2010) with a significant effect on the quality of life, high rates of morbidity and mortality and an enormous economic burden on the society (Wang et al., 2003). The pathogenesis of MDD is yet unknown although some hypotheses such as dysfunction of synaptic plasticity (Wainwright and Galea, 2013; Kuhn et al., 2014), increased apoptosis (Shelton et al., 2011; Miguel-Hidalgo et al., 2014) and aberrant autophagy (Gassen et al., 2015; Jia and Le, 2015) prevail.

Some studies have implicated deficits in the mTOR signaling system in MDD. mTOR protein levels were reported to be reduced in post-mortem prefrontal cortex of MDD patients (Jernigan et al., 2011). Chronic stress is frequently used to model MDD in rodents. In the amygdala of rats exposed to chronic stress reduced phosphorylation of components of the mTOR signaling pathway was observed (Chandran et al., 2013).

**FIGURE 1:**
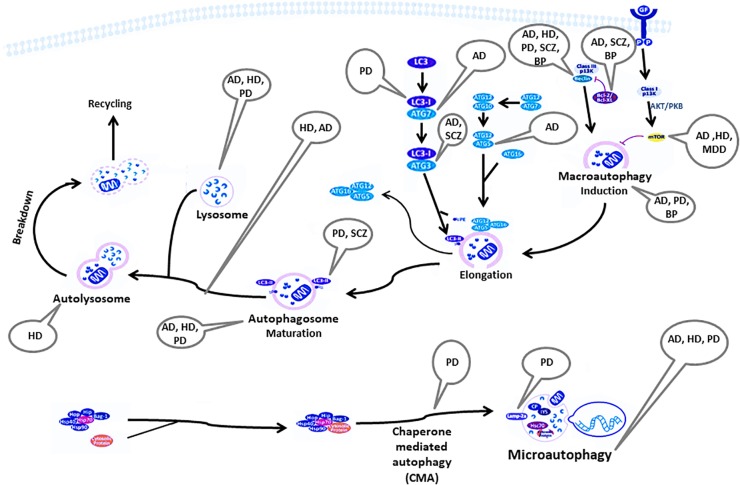
Components of the autophagy pathway reported to be deviated in neuropsychiatric disorders. AD, Alzheimer’s disease; AKT/PKB, RAC-alpha serine/threonine-protein kinase/protein kinase B; ATG, autophagy-related genes; Bag, Bcl-2 associated athanogenes; Bcl-2, B-cell lymphoma 2; BP, bipolar disorder; CP, ceruloplasmin; GF, growth factor; HD, Huntington’s disease; Hip, Hsp70-interacting protein; Hop, Hsp70-Hsp90 organizing protein; HSP, heat shock protein; IP_3_, inositol 1,4,5-trisphosphate; Lamp2a, lysosome-associated membrane protein-2; LC3, light chain 3; MDD, major depressive disorder; mTOR, mammalian target of rapamycin; PD, Parkinson’s disease; PE, phosphatidylethanolamine; SCZ, Schizophrenia.

Figure 1 summarizes the autophagy pathway and its components reported to be deviated in neuro-psychiatric disorders.

## The Potential of Autophagy-Related Therapeutic Strategies for the Treatment of Neurodegenerative and Psychiatric Disorders

As summarized above both neurodegenerative diseases and psychiatric disorders exhibit characteristics of aberrant autophagy which might be a central pathophysiological factor. Therefore, we contend that modifying autophagy pharmacologically might be an approach to prevent or to halt neurodegenerative and psychiatric disorders by potentially enhancing the removal of aggregated mutant proteins/dysfunctional mitochondria by their degradation, thus protecting from cell death/distrees. Evolving conceptualization supported by molecular, cellular and behavioral data suggests that affective disorders might be regarded as neurodegenerative ones involving plasticity- and resilience-related cascades (Bachmann et al., 2005; Einat and Manji, 2006; Racagni and Popoli, 2008; Calabrese et al., 2009). In this respect, one study suggested that protein aggregation is a common feature of neurodegenerative diseases (Taylor et al., 2002) while another one – that beyond neurodegenerative disorders protein aggregation might also be involved in affective disorders (Leliveld et al., 2008). The latter reported an increase in protein aggregates in post-mortem brains of affective disorders (MDD and BPDs) and schizophrenia patients as compared to controls.

The mTOR-dependent autophagy enhancers metformin, resveratrol, isorhynchophylline and rapamycin have all been shown to increase α-synucleine clearance and reduce cell death in various PD and AD models (Pan et al., 2009; Wu et al., 2011; Lu et al., 2012; Ng et al., 2012; Kou and Chen, 2017). Mitochondrial pyruvate carrier (MPC) is a key controller of cellular metabolism that influences mTOR activation and has recently been reported to regulate autophagy. A compound that specifically targets MPC, to reduce its activity exhibited neuroprotective and anti-inflammatory effects in several PD models (Ghosh et al., 2016).

The mTOR-independent *autophagy enhancers include: a. Trehalose*, a disaccharide present in many non-mammalian species, induces autophagy and has been shown to enhance the clearance of mutant huntingtin and α-synuclein mutants in mammalian cell cultures such as monkey kidney and human neuroblastoma cells (Sarkar et al., 2007a). Studies toward unraveling the mechanism by which trehalose regulates autophagy revealed the involvement of autophago-lysosomal components including TFEB (Settembre and Ballabio, 2011) as well as transient enlargement and permeabilization of the lysosomes. Furthermore, trehalose treatment led to transcriptional activation of autophagy-related TFEB targets (Rusmini et al., 2018). The latter corroborates our group’s report that trehalose administration to mice resulted in reduction of frontal cortex p62/beclin1 ratio suggesting enhancement of autophagy (Kara et al., 2013). *b. Ca^2+^ channel antagonists, calpains, cyclic AMP (cAMP) and Gi signaling activators* induce autophagy in a variety of cells in an mTOR-independent pathway. For example, reduction of cAMP which regulates IP_3_ levels lead to a cascade of signaling with numerous potential points where autophagy can be induced (Williams et al., 2008). c. Three *small-molecule enhancers (SMER) and inhibitors (SMIR)* were identified with the ability to enhance autophagy clearance of mutant huntingtin and A53T α-synuclein in yeast independently of rapamycin (Sarkar et al., 2007b). Antidepressants and mood stabilizers have also been reported to affect autophagy. *Lithium*, the prototype mood stabilizing drug commonly used in the treatment of bipolar patients, affects neurons at an array of parameters including neuronal growth factors, inhibition of oxidative stress and autophagy (Camins et al., 2009), resulting, in animal models, in neuroprotection – potentially beneficial against neurodegeneration (Sasaki et al., 2006; Bian et al., 2007). It has been shown (Williams et al., 2002; Sarkar et al., 2005) and confirmed (Sade et al., 2016) that lithium is an autophagy enhancer via inositol metabolism in several mammalian cell lines and in mouse brain. In this context lithium has also been shown to reduce neurodegeneration in Drosophila models of HD (Sarkar et al., 2008). The beneficial effect was shown to be achieved by enhancing autophagic activity through inhibition of inositol monophosphatase and inositol transporters, free inositol depletion and decreasing inositol 1,4,5-trisphosphate (IP_3_) levels (Phiel and Klein, 2001; Sarkar et al., 2005; Sarkar and Rubinsztein, 2006). It resulted in enhanced clearance of autophagy substrates like mutant huntingtin and a-synucleins (Sarkar et al., 2005, 2008). However, opposite responses of autophagy to lithium were also observed. Lithium administration to neonatal rats with hypoxia-ischemia (HI) brain injury inhibited autophagy, as reflected by lowered levels of immuno-stained LC3 accompanied by neuroprotection. In contrast, increased LC3 levels were observed in rats with HI without lithium treatment (Li Q. et al., 2010). It is unclear whether a direct lithium’s neuroprotective effect reduced the requirement for autophagy, or whether lithium directly downregulated autophagy. Attempts to elucidate whether enhanced autophagy following HI reflects a failed compensatory effect or the mechanism mediating cell death have been limited due to the lack of specificity of pharmacological inhibitors.

Lithium has also been shown to inhibit (*in vitro*) glycogen synthase kinase-3β (GSK-3β) that reduces autophagy by activating mTOR (Klein and Melton, 1996; Stambolic et al., 1996; Sarkar et al., 2008). However, we have recently shown that lithium’s inhibition of GSK-3β does not occur in lithium-treated patients (Azab et al., 2017). Additional mood stabilizers proven to cause inositol depletion (Williams et al., 2002), *valproic acid and carbamazepine*, were also reported to upregulate autophagy (Fu et al., 2010; Renna et al., 2010), reduce apoptosis and alleviate mitochondrial dysfunction in rotenone-exposed SH-SY5Y cells, a PD model (Williams et al., 2008; Hou et al., 2015).

*Ketamine*, a recently recognized rapid-acting antidepressant (Berman et al., 2000; Zarate et al., 2006b; Diazgranados et al., 2010), has been shown to activate mTOR in rat prefrontal cortex resulting in increased synaptic signaling proteins and number and function of new spine synapses (Li N. et al., 2010). A similar effect of a continued increase of mTOR phosphorylation from baseline up to 100 min following ketamine infusion was observed in peripheral blood cells of a depressed patient (Li N. et al., 2010) and in the plasma of three patients (Denk et al., 2011; Yang et al., 2013). Activation of mTOR may be expected to inhibit autophagy rather than activate it. However, except from mTOR’s role as an autophagy inhibitor, mTOR is influenced by the activity of neuronal receptors including *N*-methyl-D-aspartate (NMDA) and tyrosine kinase (Trk) B. Indeed, chronic ketamine administration had no effect on depressive-like behavior and frontal cortex autophagy in mice (Kara et al., 2017).

Conventional antidepressant drugs have also been shown to enhance autophagy via several molecular mechanisms [Alcocer-Gomez et al., 2017 (Gulbins et al., 2018 #379)]. *Amitriptyline* and *citalopram* upregulated the expression of the autophagy markers LC3B-II and Beclin1 in primary astrocytes and neurons (Zschocke and Rein, 2011). Beclin1 was increased following acute antidepressant treatment in mouse brain (Gassen et al., 2014). In a mouse model of depression, learned helplessness (LH), treatment with *fluoxetine* resulted in elevation of Beclin1 expression and mitophagy protein levels (Li et al., 2016). Exposure of glioma cells to *imipramine* inhibited PI3K/Akt/mTOR signaling, stimulated the conversion of LC3-I to LC3-II and re-distributed LC3 to autophagosomes, suggesting stimulation of autophagy progression (Jeon et al., 2011).

An add-on double blind placebo-controlled pilot phase II study of AVN-211 (3-sulfonyl-pyrazolo[1,5-a]pyrimidine), a specific antagonist of the G-protein coupled receptor 5-HT6R, showed a pro-cognitive (attention) effect in patients with schizophrenia stabilized on antipsychotic medication (Morozova et al., 2014). 5-HT6R activation increased mTOR signaling in rodent prefrontal cortex (Meffre et al., 2012). Hence, these observations raise the possibility that recruitment of mTOR by prefrontal 5-HT6R plays a role in the ruffled cognition in schizophrenia and offers a novel target for its therapy.

## Discussion

Autophagy plays a key role in maintaining cellular homeostasis and survival by promoting a clearance mechanism of mutant/misfolded proteins. Despite a dramatic progress in understanding the molecular and cellular mechanisms of autophagy in the past two decades full comprehension is still limited. Reviewed here is evidence of autophagy’s important role in neurodegenerative and mental illnesses. However, it is still unclear under which circumstances enhanced autophagy plays a role in cell death or represents a rescue mechanism with protective effects (Rubinsztein et al., 2005; Carloni et al., 2008; Koike et al., 2008). Furthermore, whether autophagy can cause cell death directly or is it a secondary effect of apoptosis remains an open question (Shintani and Klionsky, 2004). Some studies have shown that inhibition of autophagy increases neuronal survival in cases such as hypoxic/ischemic brain injury in mice and necrotic cell death in *Caenorhabditis elegans* (Yang et al., 2007; Cherra and Chu, 2008; Koike et al., 2008; Samara et al., 2008; Uchiyama et al., 2009). It should be noted though that the increased number of autophagosomes is not always an indicator of enhanced autophagy. It may also be translated as either accumulation of uncleared autophagosomes due to impaired fusion to the lysosomes, or as a dysfunction in one of the various autophagy induction pathways (Boland et al., 2008; Rubinsztein et al., 2009).

Autophagic failure consequences are different depending on the stage at which failure occurs. It might occur at the level of autophagosome formation, resulting in accumulation of discarded cargo such as misfolded proteins and/or dysfunctional mitochondria leading to a toxic cost to the neuron (Hara et al., 2006; Komatsu et al., 2006, 2007). Another failure level might be the recognition of autophagic cargo. This may cause the same corollaries as above, and depends on the extent of the recognition of dysfunction and the type of cargo. A third level of defect might arise if the autophagosomes are not cleared properly, leading to their accumulation. This might interfere with intracellular trafficking and even result in toxic outcomes as seen in AD, where the presence of APP in autophagosomes results in the production of β-amyloids (Yu et al., 2005). Hence, as summarized by Zhang et al. (2017) both ubiquitin proteasomal system (UPS) dysfunction and the autophagy-lysosomal pathway (ALP) impairment might be involved in the pathological misfolded/aggregated protein clearance. Identifying the specific level of autophagy failure is imperative for future development of therapeutic modalities.

In relation to potential therapeutic strategies for the treatment of neuropsychiatric disorders based on the intervention in autophagy lithium provides a crossroad linking autophagy, mood stabilization (an anti-depressant and an anti-manic agent) and, possibly, neurodegenerative/neurodevelopmental disorders. Lithium has been shown to enhance autophagy and to upregulate mitochondrial function, both effects mediated by inositol depletion (Sarkar et al., 2005). Enhanced autophagy may result in enhanced clearance of damaged mitochondria (mitophagy) resulting in increased mitochondrial turnover and, consequently, in upregulated mitochondrial function. Indeed, a transcriptomics study of our group found “up-regulation of mitochondrial genes in lithium treated mice and in phosphoinositide cycle-related knockout mice” (Toker and Agam, 2014; Toker et al., 2014) corroborating reports regarding mitochondrial dysfunction in bipolar patients (Wang, 2007). Hence, lithium might be considered as a treatment strategy to enhance autophagy in an mTOR-independent manner in neurodegenerative diseases such as HD (Sarkar et al., 2005). Another therapeutic option might have been rapamycin-induced mTOR inhibition. However, since mTOR inhibition affects other pathways (Ravikumar et al., 2006), and since chronic rapamycin treatment is accompanied by severe side effects (Bove et al., 2011), more specific ways to activate autophagy are required. Furthermore, it has been shown that rapamycin loses its efficacy during the progression of HD (Ravikumar et al., 2004). A different potential thinking path may be based on the finding that neuroinflammation affects brain autophagy and contributes to the progression of neurodegenerative disorders (Francois et al., 2014). This may imply a new therapeutic strategy for CNS disorders. An additional issue to be considered is genetic findings showing that proteins related to neurodegeneration, such as huntingtin, participate in autophagy as one of their physiological functions (Menzies et al., 2017). This suggests that the applicability of targeting autophagy as a whole might be limited in neurodegenerative diseases (Scrivo et al., 2018). Hence, perhaps future efforts should focus on targeting specific types and steps of the autophagic process.

When considering intervention in autophagy as an approach to overcome neuropsychiatric disorders age might be a crucial factor to be considered. In adulthood-related disorders such as neurodegenerative diseases (and certain tumors) autophagy-inducing drugs have been shown to be beneficial (Levine and Kroemer, 2008; Winslow and Rubinsztein, 2008; Rubinsztein et al., 2012). We have previously demonstrated antidepressive- and antimanic-like effect of autophagy enhancers in *adult* rodents (Kara et al., 2013, 2017; Sade et al., 2016). Perplexingly, as mentioned above, HI in the *immature brain* resulted in *increased autophagy in injured neurons* (Li Q. et al., 2010). In addition the authors demonstrated that lithium protects neurons derived from postnatal day 8–9 of rodents by inhibiting apoptosis and autophagy. In this respect it is interesting to note that a recent clinical trial (Yuan et al., 2018) found that low dose lithium (well established to enhance autophagy in cells in culture and adult brain) improved cognitive performance and adaptive behavior in children aged 4–11 years old with intellectual disability. As information regarding autophagy in oligodendrocytes in intellectual disability is still missing, it is imperative, both at the basic/translational and at the applicative level, to investigate whether there are age-related differences in the mechanism(s) of lithium-induced beneficial outcomes via the drug’s effect on autophagy.

In conclusion, stimulation of autophagy in neuropsychiatric disorders may be a neuroprotective strategy, keeping in mind avoiding excessive process which might be destructive. It is essential to take into account that modifying autophagy may lead to diverse consequences and interfere with mechanisms yet unraveled. By revealing the molecular mechanisms involved in autophagy and the role of this process in neuronal life and death pathways it might be possible to either inhibit or stimulate autophagy for therapeutic purposes.

## Author Contributions

OD and TB-Y ran the literature search and prepared the first draft. GA instructed OD and TB-Y, and brought the manuscript for submission.

## Conflict of Interest Statement

The authors declare that the research was conducted in the absence of any commercial or financial relationships that could be construed as a potential conflict of interest.
